# Integrative immune analysis in patients with leprosy reveals host factors associated with mycobacterial control

**DOI:** 10.1016/j.ebiom.2025.105855

**Published:** 2025-07-18

**Authors:** Anouk van Hooij, Krista E. van Meijgaarden, Marufa Khatun, Santosh Soren, Kimberley Walburg, Khorshed Alam, Abu Sufian Chowdhury, Colette L.M. van Hees, Jan Hendrik Richardus, Annemieke Geluk

**Affiliations:** a Deptartment of Infectious Diseases, LUCID, Leiden University Medical Center, the Netherlands; b Research Program, The Leprosy Mission International Bangladesh, Nilphamari, Bangladesh; c Department of Dermatology, Erasmus Medical Center, Rotterdam, the Netherlands; d Department of Public Health, Erasmus MC, University Medical Center Rotterdam, Rotterdam, the Netherlands

**Keywords:** Mycobacterial growth inhibition assay, Spectral flow cytometry, Leprosy, Immunophenotyping, BCG, *Mycobacterium leprae*

## Abstract

**Background:**

Leprosy is a debilitating, chronic infectious disease, ranking second after tuberculosis in the order of severe human mycobacterial diseases. If timely treatment is not initiated, infection with its causative agent, *Mycobacterium leprae*, can result in severe nerve damage leading to life-long disabilities. Host immunity largely dictates the spectral disease presentation, ranging from multi- to paucibacillary. Studying the host response to *M. leprae* is, however, complicated by the inability to culture this mycobacterium *in vitro*. Immune correlates of protection in persons at risk of leprosy are, therefore, essentially unknown.

**Methods:**

To identify host factors related to mycobacterial control, functional mycobacterial growth inhibition assays combined with extensive immunophenotyping by spectral flow cytometry were performed for patients with leprosy and their contacts. This integrative approach merged sampling of peripheral blood mononuclear cells in low resource areas with immune-analysis using cutting edge technology.

**Findings:**

In contrast to the current dogma, no intrinsic differences in mycobacterial control *in vitro* between patients with high and low bacillary loads were observed. Immunophenotyping at consecutive levels revealed a significant link between the induction of chemokines to mycobacterial antigens and expression of CXCR3 and CCR4 on adaptive immune cells in contacts controlling *M. leprae* infection.

**Interpretation:**

These results offer more detailed insights into protective immunity against *M. leprae* and define host factors associated with bacterial control, fuelling improved diagnosis and treatment of leprosy.

**Funding:**

10.13039/501100009425Q.M. Gastmann-Wichers Foundation, the 10.13039/501100021734Leprosy Research Initiative & the 10.13039/100017714Turing Foundation (ILEP#: 707.19.02), R2STOP; the Leprosy Mission Great Britain.

**Research in context**

**Evidence before this study**

Patients with leprosy present diverse clinical symptoms and bacterial burden, requiring well-trained clinicians to recognise the often-misdiagnosed signs and symptoms. The clinical spectrum of leprosy is determined by host immunity to *Mycobacterium leprae*. The current dogma is that paucibacillary patients mount an effective cell-mediated immune response to *M. leprae*, whereas multibacillary patients display a humoural immune response characterised by numerous *M. leprae*-specific antibodies. The majority of contacts exposed to *M. leprae* will never develop disease. It is, however, unknown which immune response protects against development of leprosy in these individuals. Studying host immunity to *M. leprae* is complicated by the inability to culture this mycobacterium in the laboratory. Mycobacterial growth inhibition assays (MGIAs) provide a functional assay to assess the ability of immune cells to control mycobacterial growth. The previous application of Bacillus Calmette-Guérin (BCG) in the MGIA as a more accessible mycobacterial surrogate for *Mycobacterium tuberculosis*, which is closely related to the non-culturable *M. leprae*, renders this assay promising to study the control of mycobacterial growth in *M. leprae* infected individuals.

**Added value of this study**

The here described integrative approach, combining sampling of immune cells in low-resource settings with cutting-edge immunological techniques, proved feasible and opens up opportunities to unravel the protective host response to *M. leprae*. The obtained results provided a comprehensive overview of antimycobacterial host immunity in patients and contacts. In contrast to their response to *M. leprae in vivo*, multi- and paucibacillary patients did not differ in their capacity to control mycobacterial growth in the MGIA. Moreover, the extensive immunophenotyping identified host immune cells associated with the different poles of the leprosy spectrum beyond the current dichotomous dogma of either cellular or humoural immunity. Expression of chemokine receptors CXCR3 and CCR4 on adaptive immune cells and a strong induction of chemokines in response to BCG in contacts was demonstrated, providing key insights to identify immune correlates of protection for leprosy disease.

**Implications of all the available evidence**

The MGIA, reported we believe for the first time in leprosy research, provides a functional assay to assess the host response to closely related mycobacteria that can be cultured in the laboratory, in contrast to *M. leprae*. The definition of host factors associated with bacterial control in contacts offers potential leads for future research, aiming to further unravel the protective immune response to *M. leprae*. Elucidating this protective response will aid to improve current diagnostic techniques and creates prospects to assess the efficacy of prophylactic interventions.

## Introduction

Leprosy is a poverty-related, neglected tropical disease, mainly affecting individuals in low-income and middle-income countries (LMICs),^1^^,^^2^ but is also on the rise in the southeastern United States.^3^, ^4^, ^5^ This chronic infectious disease, caused by *Mycobacterium leprae* and/or *Mycobacterium lepromatosis*, presents with diverse clinical symptoms, including skin lesions, nerve and tissue damage. Antibiotic treatment is available but cannot restore the nerve damage caused by the unique ability of its causative pathogens to infect Schwann cells.^6^ Today, more than 4 million people suffer from permanent leprosy-associated disabilities and handicaps, heavily impacting their daily life.^7^ As host immunity against *M. leprae* dictates presentation of the leprosy spectrum, patients with leprosy are classified based on the cell-mediated immune (CMI) response to *M. leprae*.^8^ Strong CMI induced by Th1 cells is observed in tuberculoid patients (TT) with few detectable bacilli contained in granulomas. The disseminated form of the disease with high bacterial loads, lepromatous leprosy (LL), is characterised by a Th2-mediated weak CMI response. Between these two poles of the spectrum lie the clinically unstable borderline tuberculoid (BT), midborderline (BB) and borderline lepromatous (BL) patients, with decreasing CMI and increasing amounts of bacilli and lesions, respectively. The humoural immune response displays an opposite pattern, as high antibody titres to *M. leprae* antigens are predominantly observed in LL/BL patients.

Detection of antibodies directed against a major component of the *M. leprae* cell wall, phenolic glycolipid I (PGL-I), can be applied as adjunct diagnostic for LL/BL patients^9^, ^10^, ^11^ and permits treatment monitoring of patients with high bacillary loads.^12^, ^13^, ^14^ Although various mycobacteria produce PGLs, the PGL-I trisaccharide is highly specific for *M. leprae* and responsible for the unique Schwann cell tropism.^6^^,^^15^^,^^16^ Despite decades-long studies by multiple research groups on their diagnostic application,^17^, ^18^, ^19^ the exact functional role of anti-PGL-I antibodies remains unknown. The high antibody titres in severe disseminated forms (LL/BL) and virtual absence in the self-limited disease (BT/TT) implicates that these antibodies do not confer protection. This strong relation between host immunity and the immunopathological spectrum renders leprosy a unique infectious disease, also offering potential as a model for other diseases.^20^^,^^21^ In fact, not only was leprosy the first human disease for which the relevance of the Th1/Th2 dualism was demonstrated,^22^ it also enabled the identification of regulatory T cells^23^ and yielded initial evidence for HLA-disease association.^24^^,^^25^

Skin lesions of patients with leprosy are commonly evaluated to study mechanisms of mycobacterial control at the site of infection.^26^^,^^27^ However, this approach does not allow to study the anti-mycobacterial response of infected individuals lacking lesions or other clinical symptoms. At greatest risk of contracting *M. leprae* infection and subsequently developing disease are household contacts of patients.^28^, ^29^, ^30^ Although the vast majority of these contacts will never progress to disease,^31^ immune correlates of protection to identify those able to control *M. leprae* infection without developing clinical signs are not available. Due to the inability to culture *M. leprae in vitro*^31^ and the similar response to *M. leprae* antigens observed in infected contacts and patients with few bacilli such correlates are difficult to determine.^32^, ^33^, ^34^ The immune response that confers protection against leprosy disease, thus, remains to be elucidated.

It is evident from the immunopathology across the leprosy spectrum, that the presence of *M. leprae* indicates the individual's inability to cope with the infection *in vivo*. The host's capacity to reduce mycobacterial growth *in vitro* can be quantified by the mycobacterial growth inhibition assay (MGIA),^35^ in which potent growth control of Bacillus Calmette-Guérin (BCG) was observed in individuals recently exposed to *M. tuberculosis*.^36^ BCG is the live attenuated vaccine form of *Mycobacterium bovis* provided to neonates to protect against tuberculosis, and this vaccine also protects against leprosy,^37^ though the protective effect wanes over time and varies between populations.^38^, ^39^, ^40^ The application of BCG in the MGIA as a more accessible surrogate for *M. tuberculosis*,^41^^,^^42^ which is closely related to the non-culturable *M. leprae*,^43^ renders the MGIA a promising assay to study the control of mycobacterial growth in *M. leprae* infected individuals.

To identify host factors related to mycobacterial control in patients with leprosy and their contacts, the capacity of peripheral blood mononuclear cells (PBMCs) to limit BCG growth *in vitro* was assessed. PBMCs used in this assay were isolated by local lab staff in Bangladesh, providing proof of feasibility to perform high-complexity immunological methods on samples processed in leprosy endemic areas under low resource conditions. Extensive immunophenotyping of monocytes, NK cells, CD4+ T cells, CD8+ T cells and B cells was performed on the same PBMCs by spectral flow cytometry.^44^ Finally, cyto-/chemokine production by these PBMCs after BCG stimulation was determined as well. The combined approach allowed integrative, consecutive analysis of the capacity to control mycobacterial growth with immunophenotype and PBMC responsiveness. This study goes beyond the well-defined Th1/Th2 dichotomy between BT/TT and LL/BL patients that is adhered to for decades, providing additional host factors associated with the control of *M. leprae* infection in contacts of patients with leprosy.

## Methods

### Study participants

Participants including patients with leprosy (n = 46) and household contacts (n = 17) were recruited in leprosy endemic districts in Bangladesh (Nilphamari, Rangpur, Panchagar, and Thakurgaon) in July 2019 (Supplementary Table S1). The leprosy prevalence was 0.9 per 10,000 and the new case detection rate 1.18 per 10,000 (Rural health program, the leprosy mission Bangladesh, yearly district activity report 2018). In Bangladesh, patients were sampled in the field and PBMCs isolated at a low resource laboratory infrastructure requiring shipment on dry ice to LUMC within 3 days after sample processing. Thus, patients were sampled before (n = 17) or after receiving multidrug therapy (MDT) for one (n = 1), six (n = 1) or fifteen (n = 1) days and one (n = 4), two (n = 17), three (n = 4) or four (n = 1) months. In addition, five patients with leprosy diagnosed in the Netherlands between 2009 and 2020 were sampled before MDT, all of which were immigrants from leprosy endemic areas. The country of sampling is indicated in Supplementary Table S1. Both males and females were included in this study, with a similar percentage of females in the patient (41%) and contact group (37.5%). Sex was self-reported by study participants.

Leprosy was diagnosed based on clinical, histological and bacteriological observations and classified using the Ridley-Jopling classification system.^8^ In Bangladesh, Ridley–Jopling classification was based on the clinical presentation of skin and nerve lesion morphology and additional slit-skin smears were used to determine the bacterial index (BI).^45^ In the Netherlands, the presence of *M. leprae* in biopsies was determined by RLEP qPCR.^29^ Patients with detectable *M. leprae* in biopsies were also classified as BI positive for this study.

In Bangladesh, as in other tuberculosis endemic areas, BCG vaccination is provided to neonates as part of the national immunisation programme. The relationship with the index case was known for each contact (Supplementary Table S1) and none of them received either BCG or a single dose of rifampicin as post-exposure prophylaxis at least 3 years prior to inclusion.

### Ethics

This study was performed according to the Helsinki Declaration (2008 revision). Ethical approval of the study was obtained from local ethical boards in Bangladesh, the National Research Ethics Committee (Bangladesh Medical Research Council) (Ref no. BMRC/NREC/2010-2013/1534; BMRC/NREC/2016-2019/214), and the Netherlands (MEC-2012-589).

Participants were informed about the study objectives, the samples and their right to refuse to take part or withdraw from the study without consequences for their treatment. Written informed consent in local language was obtained before enrolment. All patients received treatment according to national guidelines.

### Blood collection and PBMC isolation

Whole venous blood was collected in two 8 ml heparinized Vacutainer® cell preparation (CPT™) tubes (Becton Dickinson, Erembodegem, Belgium). Within 24 h of collection, PBMCs were isolated according to the manufacturer's protocol. 2 ml plasma was collected during the procedure and frozen at −20 °C. PBMCs were frozen in foetal bovine serum/10% DMSO and stored in liquid nitrogen. PBMCs isolated at the local laboratory in Bangladesh were kept for 3 day at −80 °C before shipment on dry ice to LUMC.

### Mycobacterial growth inhibition assay

MGIAs were performed at the BSL2 lab at the LUMC. The optimised ‘in tube’ MGIA Euripred protocol was used, as described previously.^36^^,^^41^^,^^46^^,^^47^ MGIAs were run in three independent experiments. 10% BD MGIT™ PANTA™ Antibiotic Mixture was added to all PBMC cultures during mycobacterial growth inhibition assays. BD MGIT™ PANTA™ Antibiotic Mixture does not affect the growth of mycobacteria. Donors were randomly selected, with an even distribution of LL/BL patients, BT/TT patients and HC per experiment. Cryopreserved PBMCs were thawed and rested in a tube with RPMI (Gibco life sciences, Thermo Fisher Scientific Inc., Bleiswijk, the Netherlands) supplemented with glutamax (Gibco) and 10% FBS (Hyclone, Thermo Fisher Scientific Inc.) (=R10 medium) at a concentration of 2 × 10^6^ cells/ml for 2 h in the presence of benzonase (20 U/ml, Merck, Amsterdam, the Netherlands). After resting, cells were washed with R10 medium and counted with a Casy Cellcounter (Roche, Woerden, the Netherlands). 1 × 10^6^ PBMCs were co-cultured for 4 days in RPMI supplemented with glutamax and 10% autologous human serum and 10% PANTA with 2.6 logCFU (±0.04 SD) of the *M. bovis* BCG Pasteur (P3) strain on a rotator in a 37 °C humidified CO_2_ incubator in a final volume of 600 μl. All samples were run in duplicates. After 4 days, 100 μl supernatant was harvested and stored for future analysis and the remaining 500 μl per sample were transferred to a PANTA/Enrichment supplemented MGIT tube (Becton Dickinson, Erembodegem, Belgium) and placed in a BACTEC MGIT 960 system (Becton Dickinson, Erembodegem, Belgium) until time to positivity (TTP) was reached. All tubes included in the analysis were checked visually for possible contamination. Since the inoculum of 2.6 logCFU BCG is expected to reach TTP after more than 250 h, samples reaching positivity within 100 h were considered contaminated and deleted as false positive. As a control for the BCG inoculum all experiments included a serial dilution (10^7^–10^2^) of the BCG stock for time to positivity in PANTA/Enrichment supplemented MGIT tubes and plating on Middlebrook 7H10 agar plates, supplemented with 10% OADC (Becton Dickinson, Erembodegem, Belgium) for CFU determination.

### Measurement of cyto-/chemokine production after BCG stimulation

As previously described,^41^ a 40-plex chemo-cytokine Bio-plex assay (Luminex®) was performed according to manufacturer's instructions (Bio-Rad, Veenendaal, the Netherlands) on supernatants collected after 96 h of BCG stimulation (from the functional MGIA). Samples were analysed on a Luminex 200 system with Bio-Plex manager software (v6.1).

### Spectral flow cytometry

Priority was given to the functional assay, but all samples with at least 1 × 10^6^ cells remaining after the MGIA were also subjected to detailed phenotyping by spectral flow cytometry using a 32-colour panel (Supplementary Table S2). The procedure and flow-cytometry panel used has been described previously.^44^ In short, a minimum of 1 × 10^6^ rested cells were incubated with cell viability dye in PBS, washed in PBS/0.1% BSA (Sigma, Merck Life Science NV, Amsterdam, the Netherlands) followed by blocking Fc receptors with 5% human serum in PBS for 10 min. Cells were washed twice and subsequently stained for chemokine receptors for 30 min at 37 °C. After washing, cells were incubated with all other surface markers at 4 °C for an additional 30 min. Cells were washed twice and fixed for 15 min at RT with Fix A (Nordic MUbio, Susteren, the Netherlands). After addition of FixA, cells were washed and subsequently stained for intracellular markers in perm buffer B for 30 min at 4 °C. Afterwards cells were washed twice and fixed with 1% paraformaldehyde (Pharmacy LUMC, Leiden, the Netherlands). After 10 min, cells were washed once more and stored at 4 °C for acquisition on a 5 L Cytek®Aurora (Cytek Biosciences, Fremont, CA, USA) the following day at the LUMC flow core facility (https://www.lumc.nl/research/facilities/fcf/).

### Anti-PGL-I antibody ELISA

ELISAs were performed to determine the presence of IgM and IgG antibodies against the immunodominant trisaccharide part of the *M. leprae* phenolic glycolipid antigen coupled to human serum albumin (HSA). Synthetic PGL-I was obtained through the Biodefense and Emerging Infections Research Resources Repository (http://www.beiresources.org/TBVTRMResearchMaterials/tabid/1431/Default.aspx).

Clear Flat-Bottom Immuno Nonsterile 96-Well PolySorp NUNC plates (Thermofisher) were coated with 200 ng synthetic PGL-I (Product#:19347 www.beiresources.org) in 0.1 M Na_2_CO_3_/NaHCO_3_ coating buffer, pH 9.6. 0.1% BSA in coating buffer was used as background control. Coated plates were incubated overnight at 4 °C and subsequently washed three times with 200 μl PBS/0.05% Tween 20. Per well, 200 μl PBS/1% BSA/0.05% Tween 80 was added and incubated at room temperature for a minimum of 1 h. After incubation, the blocking buffer was removed and 50 μl 1:400 diluted plasma was added to a well coated with synthetic PGL-I and 50 μl to a well with 0.1% BSA for each sample. After 2 h of incubation at room temperature, the wells were washed three times as described above. Per well, 50 μl anti-IgM-HRP (1:8000; Sigma A6907; RRID: AB_258318) or anti-IgG-HRP (1:4000; Sigma A0170; RRID: AB_257868) in PBS/0.05% Tween 20 was added and incubated at room temperature for 2 h. After washing the ELISA plate four times with the procedure described above, 50 μl 3,3′,5,5′-Tetramethylbenzidine (TMB, Thermo Fisher Scientific, Bleiswijk, the Netherlands) was added allowing the colour reaction to start. The reaction was stopped after 10 min by adding 50 μl 1 M H_2_SO_4_. Absorbance was determined at a wavelength of 450 nm (Spectramax i3x microplate reader).

### Data analysis

CFUs were determined by counting colonies using ImageJ software, after scanning the plates on a Canon Scanner 9000 F. CFUs were converted to logCFU and plotted against TTP (Supplementary Figure S1). Linear regression analysis was applied (GraphPad Prism software v9.3.1), all samples were transposed and data plotted as logCFU. Samples with a logCFU < 2.59 (lower limit of the CI of the median of the controls) were considered to control the growth of BCG in the MGIA. Good control was defined as samples with more than 1 log reduction compared to the median of controls (<1.59). LogCFU values between 1.59 and 2.59 were described as intermediate control.

Analysis of the spectral flow cytometry data was performed using OMIQ analysis software (www.omiq.ai). Datafiles were cleaned by setting a time gate and removal of doublets. The monocyte and lymphocyte populations were selected using forward versus side scatter gating. Within this gate, monocyte (CD3−/CD14+), NK cell (CD3−/CD56+), T cell (CD3+/CD14−), and B cell (CD3−/CD19+) populations were identified by manual gating (Supplementary Figure S2).

Further in-depth analysis was performed using unsupervised dimension reduction and clustering methods. Uniform Manifold Approximation and Projection (UMAP) dimension reduction (Neighbours: 5; Minimum distance: 0.01; Components: 2; Metric: euclidian; Learning rate: 1; Epochs: 200) was applied to a subsample of monocytes (10.000 cells per sample or maximum available cells), CD4+ and CD8+ T cells, NK cells and B cells (50.000 cells per sample or maximum). FlowSOM (xdim:10; ydim:10; rlen: 10; Distance metric: Euclidian) was applied to identify metaclusters (Elbow metaclustering) within these populations.^48^ For further downstream analysis, populations with less than 100 cells were excluded. Mean fluorescence intensity (MFI) of each of the markers in the 32-colour panel was determined per metacluster. Data sets were further exported for univariate analysis in GraphPad Prism (v10.2.3). Individuals with missing data were not incorporated in the respective analysis.

### Statistics

p-value calculation by Mann–Whitney U test (two groups) or Kruskal–Wallis with Dunn's multiple test correction (multiple groups), Spearman correlation and linear regression were performed using Graphpad Prism (v10.2.3). Heatmaps with clustering based on average linkage were generated using heatmapper.^49^ Correlation matrix was computed using Rstudio (v2023.12.1) and principal component analysis (PCA) biplots were generated using Orange data mining (v3.36.2), an open source machine learning tool.^50^

### Role of funders

Funders did not play a role in the study design, data collection, data analysis and interpretation of this study.

## Results

### Patients with high and low bacterial load show similar capacity to control mycobacterial growth *in vitro*

**Fig. 1:**
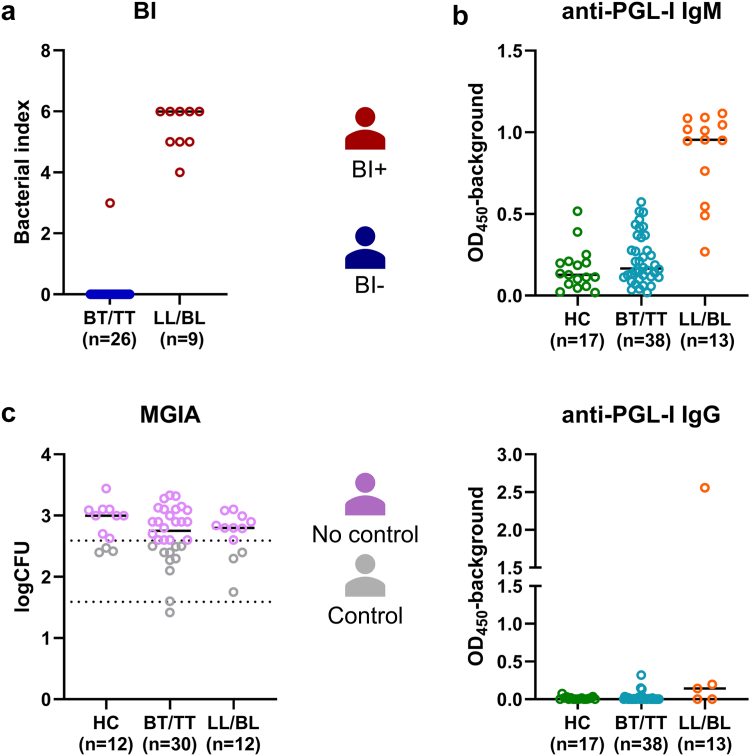
**Patients with a high and low bacterial load show similar capacity to control mycobacterial growth *in vitro*.** (a) Bacterial index (*y*-axis) of lepromatous (LL/BL; n = 9) and tuberculoid (BT/TT; n = 26) leprosy patients. (b) Anti-PGL-I IgM and IgG levels determined by ELISA (OD_450_-background) in patients with leprosy (LL/BL (n = 13) and BT/TT (n = 38)) and their household contacts (HC; n = 17). (c) Result of the functional mycobacterial growth inhibition assay of patients with leprosy (LL/BL (n = 12) and BT/TT (n = 30)) and HC (n = 12). Dashed lines indicate the lower limit of the confidence interval (CI) of the median of the controls, and the 1 log reduction compared with this median. Samples above the CI of median lacked control of mycobacterial outgrowth and samples with more than 1 log reduction had good control.

To study the functional antimycobacterial response in patients with leprosy and their contacts (Supplementary Table S1), MGIAs were performed to assess the capacity of PBMCs in combination with autologous plasma to control BCG growth *in vitro*. The MGIA protocol duration does not allow to include viable *M. leprae*, which does not grow *in vitro* and would not survive the 4 days of co-culture with PBMCs. BCG was therefore applied as a closely related surrogate. In concordance with their Ridley–Jopling classification,^8^ LL/BL patients have a significantly higher *M. leprae* load than BT/TT patients and higher *M. leprae*-specific anti-PGL-I antibody levels, particularly anti-PGL-I IgM (Fig. 1a and b). These two parameters are characteristic of patients unable to control *M. leprae in vivo*. However, LL/BL patients (n = 12), BT/TT patients (n = 30), and contacts (n = 12) similarly controlled BCG growth *in vitro* (Fig. 1c). Good control (>1 logCFU reduction) was observed for PBMCs of two patients with leprosy and none of the contacts. Intermediate control was observed in three LL/BL patients (25%), eight BT/TT patients (27%), and three HC (25%). Thus, the functional antimycobacterial response to BCG does not differ significantly between patients with high and low bacterial loads, indicating that *in vivo* control of *M. leprae* does not parallel with *in vitro* capacity to control BCG.

### Analysis pipeline: immunophenotyping of PBMCs by zooming in on consecutive levels

To further unravel the host immune response to BCG and *M. leprae*, extensive immunophenotyping of PBMCs by spectral flow cytometry^44^ was performed. The 32-colour panel covered major lineage immune cells, chemokine receptors and cytokines. Individuals were stratified by the available metadata into the following groups (Fig. 1): (i) LL/BL patients (n = 12), BT/TT patients (n = 37), and contacts (n = 17) to designate the leprosy status *in vivo*. Contacts were monitored for signs and symptoms of leprosy during five years. None of the included contacts developed disease during this follow-up period, as could be expected based on the leprosy incidence in contacts in the study areas and the number of contacts sampled, indicating that these individuals are able to cope with *M. leprae* after exposure. (ii) BI positive (n = 14) and BI negative (n = 35) to designate the individual's ability to cope with infection *in vivo* based on the bacterial load. (iii) Individuals with (n = 16) or without BCG growth control (n = 35) based on the logCFU obtained by MGIA to designate mycobacterial control *in vitro*.

**Fig. 2:**
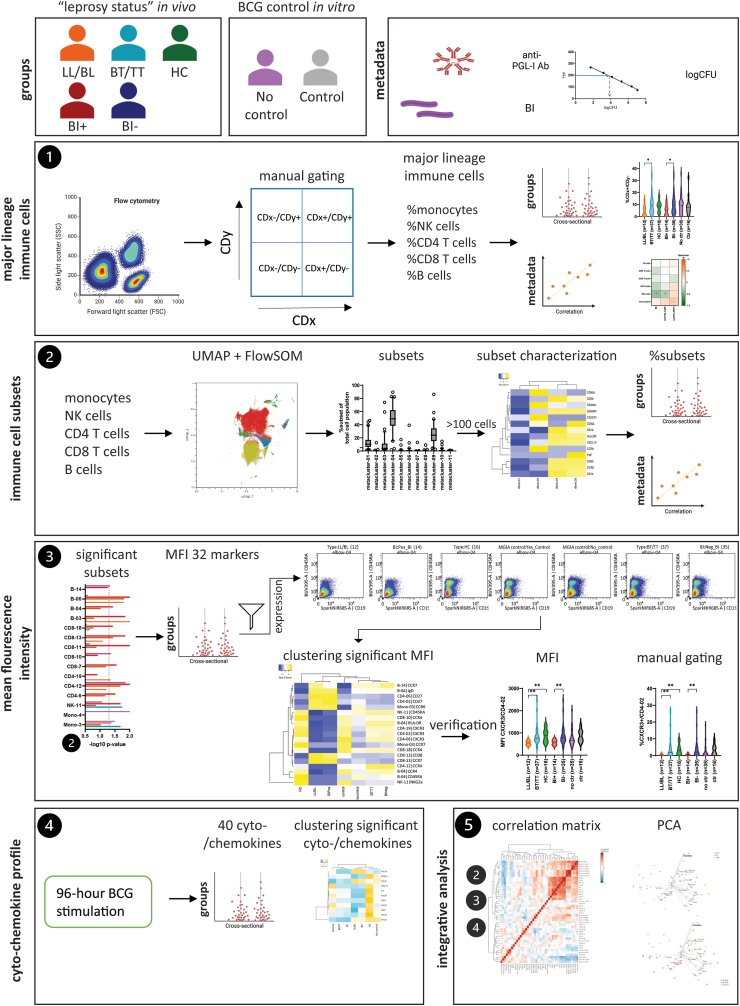
**Analysis pipeline: Immunophenotyping of PBMCs at multiple levels.** Groups: individuals were stratified based on “leprosy status” *in vivo* and BCG control *in vitro*. Abbreviations: lepromatous (LL/BL) patients, tuberculoid (BT/TT) patients, household contacts of patients with leprosy (HC), bacterial index positive (BI+) or negative (BI−) patients. Metadata: numerical metadata used in the analysis pipeline as shown in Fig. 1: anti-phenolic glycolipid-I IgM levels determined by ELISA, BI determined by acid fast staining of slit-skin smears ranging from 0 to 6, and log colony forming units (logCFU) of BCG. 1. Major lineage immune cells: The percentage of monocytes, NK cells, CD4+ T cells, CD8+ T cells, and B cells determined by manual gating. Percentages were cross-sectionally compared between groups and correlated with the available metadata. 2. Immune cell subsets: Uniform Manifold Approximation and Projection (UMAP) dimension reduction was performed on the five major lineage immune cells [1]. To identify subsets (metaclusters) based on the marker expression within these populations FlowSOM was applied. Populations >100 cells were included in the analysis and marker expression of these subsets was visualised by clustered heatmaps. Percentages of the subsets were cross-sectionally compared between groups and correlated with the available numerical metadata. 3. MFI (mean fluorescence intensity): The quality of significant (∗∗) immune cell subsets [2] was assessed by comparing the MFI of markers expressed within each subset between groups. Concatenated dot plots for the seven groups were assessed to check the expression level (high resolution images in Supplementary Figure S5). A clustered heatmap was generated to visualise the median MFI per group. To verify this approach, percentages of cells expressing significantly different markers within the CD4+ subset were determined by manual gating and cross-sectionally compared between groups. 4. Cyto-chemokine profile: Production of cyto-/chemokines by PBMCs after 96 h of stimulation with BCG. A clustered heatmap was generated to visualise the levels of significantly different cyto-/chemokines between groups. 5. Integrative analysis: Integrative analysis of significant immune cell subsets [2], MFI [3] and cyto-/chemokines [4]. A correlation matrix was computed to determine association between the results obtained in [2–4]. Significant clusters within the correlation matrix were visualised by principal component analysis (PCA) biplots.

Using these group-classifications, the immunophenotypes were analysed by zooming in on multiple consecutive levels (Fig. 2). First, quantitative analysis of major lineage immune cells was performed. The percentages of monocytes, NK cells, CD4+ T cells, CD8+ T cells, and B cells were determined by manual gating, and cross-sectionally compared between groups as well as correlated with the available numerical metadata (anti-PGL-I antibody levels, BI and logCFU). Second, immune cell subsets of these monocyte-, NK cell−, CD4+ T cell−, CD8+ T cell−, and B cell populations were identified by unbiased UMAP dimension reduction. FlowSOM was applied to identify cell subsets (metaclusters) based on marker expression patterns. Subsets containing minimally 100 cells were included in the analysis and marker expression of subsets was visualised by clustered heatmaps. Percentages of the subsets were cross-sectionally compared between groups and correlated with the available numerical metadata. Thirdly, the significant subsets were analysed further by comparing the mean fluorescence intensity (MFI) of markers expressed within each subset between groups. A clustered heatmap was generated to visualise the median MFI per group. To verify this approach, percentages of cells expressing significantly different markers within the CD4+ T cell subset were determined by manual gating. Fourthly, production of 40 cyto-/chemokines by PBMCs of the same individuals after 96 h of stimulation with BCG was assessed. A clustered heatmap was generated to visualise the levels of significantly different cyto-/chemokines between groups. Finally, these results were combined by an integrative analysis of significant immune cell subsets, MFI, and cyto-/chemokine levels. A correlation matrix was computed to determine associations and significant clusters within the correlation matrix were visualised by PCA biplots. The results of this consecutive analysis pipeline are described per level in the next paragraphs.

### High bacillary load in patients with leprosy is associated with a low percentage of total NK cells

**Fig. 3:**
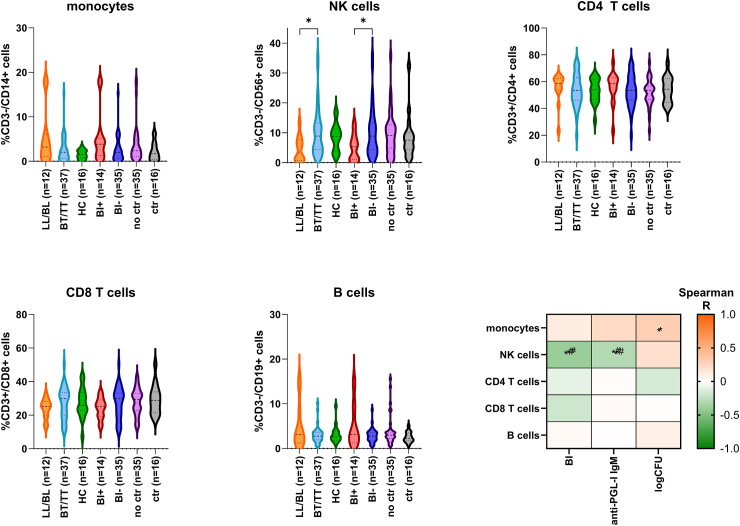
**Distribution of major immune cell subsets in patients with leprosy.** The percentage of major lineage immune cells was determined for each individual by manual gating. Violin plots show the percentage observed per group: lepromatous leprosy patients (LL/BL; n = 12); tuberculoid leprosy patients (BT/TT; n = 37); household contacts of patients with leprosy (HC; n = 16); BI positive patients (BI+; n = 14); BI negative patients (BI−; n = 35); Individuals without (no Ctrl; n = 35) or with BCG control (Ctr; n = 16) *in vitro*. Dashed and dotted lines indicate the median and quartiles, respectively. p-values were determined by Mann–Whitney U test (2 groups) or Kruskall–Wallis test with Dunn's correction for multiple testing (3 groups). ∗p-value < 0.05. Correlation of the percentage of major lineage immune cells with bacterial index (BI), anti-PGL-I IgM antibody levels and logCFU determined by MGIA was visualised in a heatmap. A negative correlation (Spearman R) was indicated by green and a positive correlation by orange. ∗ p-value < 0.05; # significant linear regression.

The percentage of major lineage immune cells was determined for each individual by manual gating (Supplementary Figure S2). Cross-sectional analysis identified the lowest percentage of NK cells in patients without *M. leprae* control *in vivo* (Fig. 3). The numerical metadata BI, anti-PGL-I IgM, and the MGIA logCFU were included in the correlation analysis. Anti-PGL-I IgG was absent in the majority of individuals (61/68) and therefore excluded from analysis. In accordance with the cross-sectional comparison, the percentage of total NK cells correlated negatively (Fig. 3) with parameters associated with disseminated *M. leprae* infection such as BI (p = 0.029) and anti-PGL-I IgM (p = 0.015). For *in vitro* BCG control, on the other hand, no significant association with the five major lineage immune cells was observed (Fig. 3).

### Immune cell subsets expressing CCR4 are associated with mycobacterial control

**Fig. 4:**
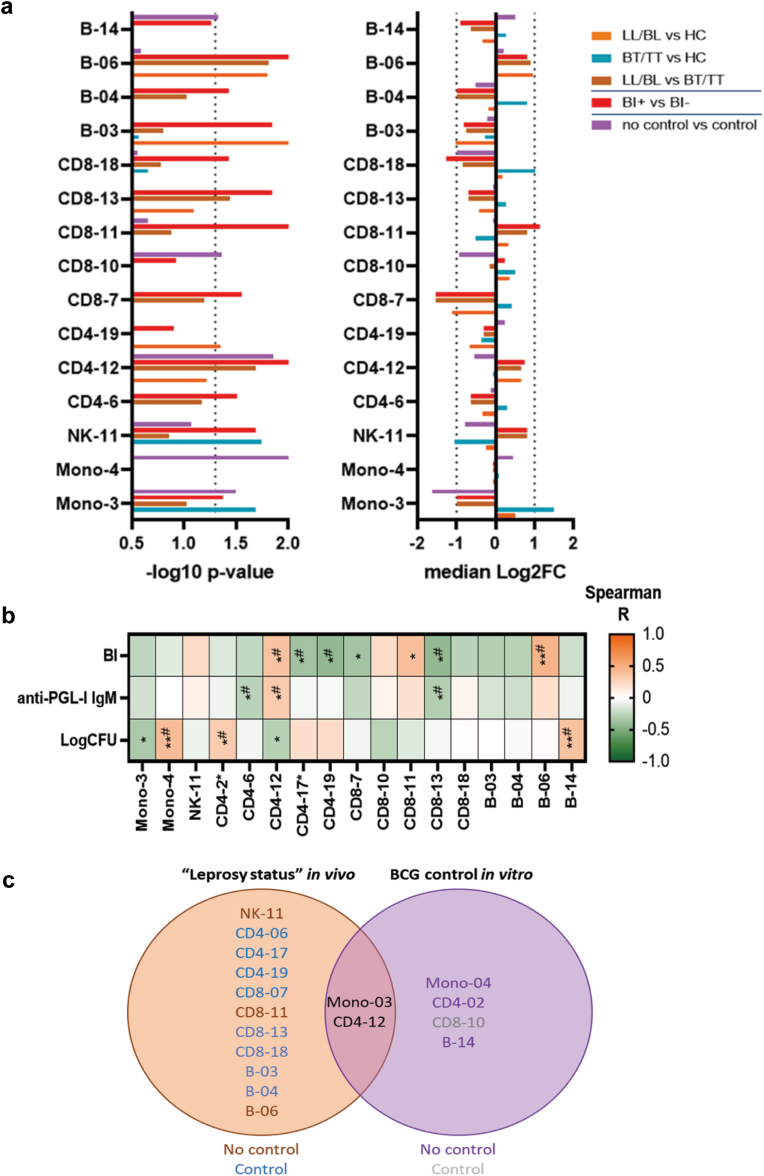
**Innate and adaptive immune cell subsets associated with mycobacterial control.** Percentages of immune cell subsets identified by UMAP dimension reduction in combination with FlowSOM were compared in lepromatous leprosy patients (LL/BL; n = 12); tuberculoid leprosy patients (BT/TT; n = 37); household contacts of patients with leprosy (HC; n = 16); BI positive patients (BI+; n = 14); BI negative patients (BI−; n = 35); Individuals without (no control; n = 35) or with BCG control (n = 16) *in vitro*. (a) –log10 p-value determined by Mann–Whitney U test (BI+ vs BI− and no control vs control) or Kruskall–Wallis test with Dunn's correction for multiple testing (LL/BL vs HC; BT/TT vs HC and LL/BL vs BT/TT). Dashed line indicates a p-value < 0.05. Median Log2 fold change indicates whether the percentage of the subsets was higher or lower in the group first mentioned (i.e., a positive log2FC indicates a higher percentage in LL/BL compared to HC). Characteristic markers per subset are indicated in Supplementary Figure S4. (b) Correlation of the percentage of significant subsets with bacterial index (BI), anti-PGL-I IgM antibody levels and logCFU determined by MGIA was visualised in a heatmap. Subsets indicated with an asterisk were identified in the correlation analysis only. A negative correlation (Spearman R) was indicated by green and a positive correlation by orange. ∗p < 0.05; ∗∗p < 0.01; # significant linear regression. (c) Venn diagram of the subsets described in A and B in relation to leprosy status *in vivo* and BCG status *in vitro*. Orange = associated with no control of *M. leprae* growth; Blue = associated with control of *M. leprae* growth. Purple = associated with no BCG control *in vitro*; grey = associated with BCG control *in vitro*.

The 32-colour spectral flow panel allowed further characterisation of monocytes, NK cells, CD4+ & CD8+ T cells, and B cells. UMAP dimension reduction in combination with FlowSOM, identified 11 monocyte subsets, 15 NK cell subsets, 19 CD4+ T cell subsets, 19 CD8+ T cell subsets, and 15 B cell subsets (Supplementary Figure S3). For 15 subsets, the percentage in which they occurred were significantly different between individuals controlling *M. leprae in vivo* (n = 10), BCG *in vitro* (n = 3) or both (n = 2) (Fig. 4a, Supplementary Figure S4). Moreover, significant correlation with BI (n = 7), anti-PGL-I IgM (n = 3) or logCFU (n = 5) was observed for 12 subsets (Fig. 4b).

The subsets Mono-03 (CD14^dim^/CCR2+/CCR4+/CD85d+) and CD4-12 (CD45RA+/CCR7+/CD27+; Supplementary Figure S3) were associated with both *M. leprae* and BCG control (Fig. 4c). The CD14^dim^/CCR4+ monocyte subset has been described previously to control BCG growth.^36^ CD4-12 expresses markers characteristic for naïve T cells and showed opposite results for *M. leprae* and BCG control: CD4-12 positively correlated with characteristics for *M. leprae* dissemination, BI and anti-PGL-I IgM levels, but was more frequently observed in individuals controlling BCG growth *in vitro* (Fig. 4).

Patients with high bacillary loads showed a higher percentage of NK-11 (CD56^hi^/NKG2a+/CD62L+), CD8-11 (CD45RA+/CD28+/CCR7+/CD27+) and B-06 (IgD+) compared to patients with low bacillary loads. Control of *M. leprae* growth *in vivo*, on the other hand, was associated with three CD4+ subsets (6, 17, and 19), three CD8+ subsets (7, 13, and 18), and two B cell subsets (3 and 4). On these subsets, CD95 and CCR4 were frequently observed: CD95 was expressed on all CD4+ & CD8+ subsets and CCR4 on all CD4+ subsets, CD8-07 and B-04. CD95 and CCR4 are thus commonly expressed on adaptive immune cell subsets of individuals able to cope with *M. leprae* infection *in vivo*.

Individuals controlling BCG *in vitro* showed a significantly lower percentage of Mono-04 (CD14^hi^/CCR2+/HLA-DR+/CD95+) and B-14 (CD45RA+/CD27+/CD20+/CD95+/HLA-DR+), and a higher percentage of CD8-10 (CD45RA+/CD28+/CCR7+/CCR4+) compared to those without control. Mono-04 had the strongest correlation with logCFU (R = 0.4; p < 0.0001), with an opposite pattern to the CD14^dim^/CCR4+ monocyte subset associated with mycobacterial control (mono-03; Fig. 4b). Hence, the quantitative analysis of immune cell subsets showed few overlap in subsets associated with control of BCG or *M. leprae*, but identified either innate or adaptive cell subsets expressing CCR4 to be associated with control of both mycobacteria.

### The level of CXCR3 and CCR4 expression is positively associated with mycobacterial control

**Fig. 5:**
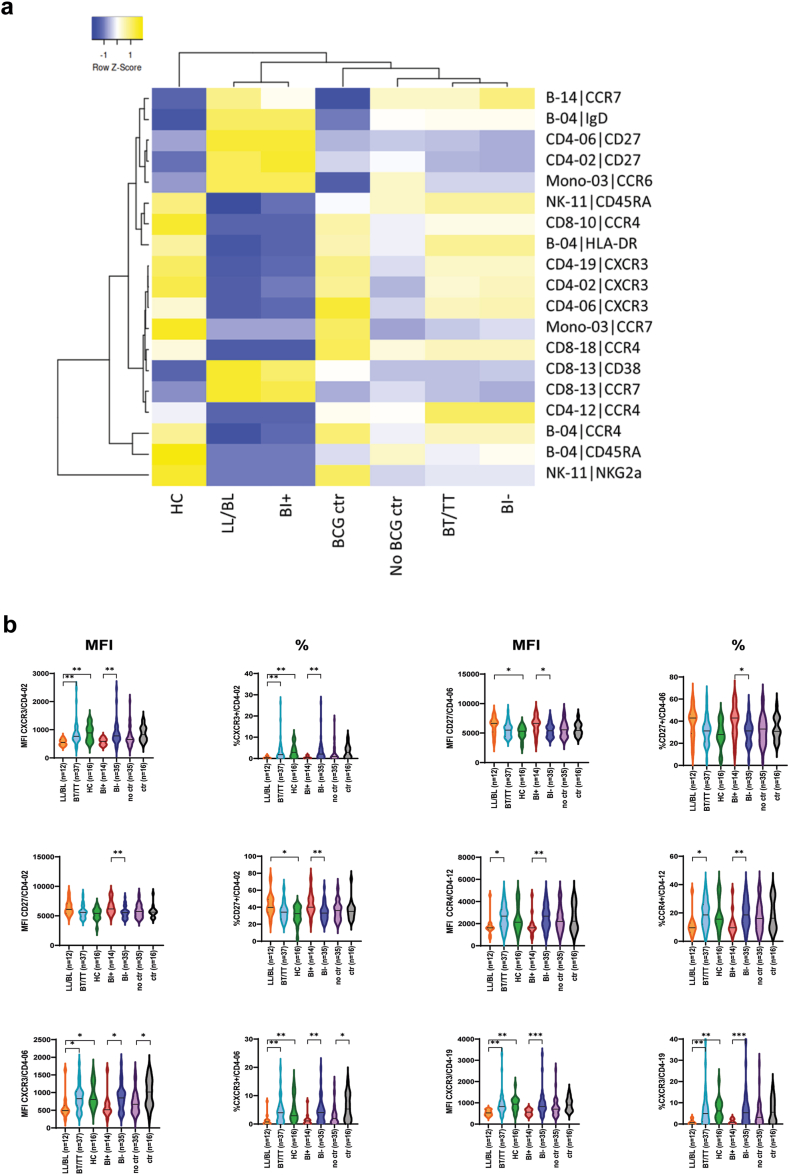
**Mean fluorescence intensity of markers within subsets.** The MFI of all markers expressed within subsets was compared in lepromatous leprosy patients (LL/BL; n = 12); tuberculoid leprosy patients (BT/TT; n = 37); household contacts of patients with leprosy (HC; n = 16); BI positive patients (BI+; n = 14); BI negative patients (BI−; n = 35); Individuals without (no ctr; n = 35) or with BCG control (ctr; n = 16) *in vitro*. (a) The median MFI per group of the significant markers (p < 0.05) was visualised in a clustered heatmap. The heatmap shows clustering based on average linkage performed by heatmapper (Babicki et al., 2016). The z-score indicates the deviation from the average level of the marker across groups, higher Z-scores are indicated in yellow and lower Z-scores in blue. (b) To validate the MFI analysis, results obtained for markers expressed on CD4+ T cell subsets were checked by manually gating CD4+ T cells expressing CXCR3, CD27 or CCR4 within the relevant subsets. Violin plots with either the MFI on the *y*-axis or the percentage of cells expressing the corresponding marker are shown for the six significant CD4+ T cells subset/marker combinations. Dashed and dotted lines indicate the median and quartiles, respectively. p-values were determined by Mann–Whitney U test (BI+ vs BI− and no control vs control) or Kruskall–Wallis test with Dunn's correction for multiple testing (LL/BL vs HC; BT/TT vs HC and LL/BL vs BT/TT). ∗p < 0.05; ∗∗p < 0.01; ∗∗∗p < 0.001.

To assess the quality of the significant immune cell subsets rather than their quantitative differences, MFI of markers expressed within subsets was compared between groups. Not only markers defining the subsets, but all markers were assessed for each significant subset (Fig. 4). Dot plots of markers identified by this analysis were evaluated to select markers with sufficient expression (Fig. 2). After filtering based on expression level, 19 significant differences in 11 unique immune cell subsets were observed (Supplementary Table S3). Median MFI of these 19 markers showed high similarity between *in vitro* BCG control and contacts controlling *M. leprae* infection (Fig. 5a). Interestingly, an almost complete inverse expression pattern was observed between the BCG control group and contacts on one hand, and patients with a high bacillary load on the other hand. Expression of CCR4 and CXCR3 on four and three immune cell subsets, respectively, was highest in groups associated with mycobacterial control, whereas CD27 on two CD4+ T cell subsets was expressed at higher levels in patients with high bacillary loads (LL/BL, BI positive). Compared to these patients, those able to limit bacterial growth (BT/TT, BI negative) express significantly more CCR4 and CXCR3 but less CD27. Regarding immunoglobulin IgD on B-04, CCR7 on B-14 and mono-03, and NKG2a on NK-11, BT/TT and LL/BL patients do show a similar expression pattern.

To verify the MFI analysis, results obtained for markers expressed on CD4+ T cell subsets were checked by manual gating. Percentages of cells expressing the markers of interest within subsets showed a similar pattern as the MFI results (Fig. 5b), thereby reflecting the results in the clustered heatmap (Fig. 5a). Thus, the corresponding marker expression pattern of leprosy contacts and *in vitro* BCG control show that intensity of expression, predominantly of chemokine receptors, is associated with mycobacterial control.

### Contacts of patients with leprosy strongly induce cyto-/chemokine production upon *in vitro* BCG stimulation

**Fig. 6:**
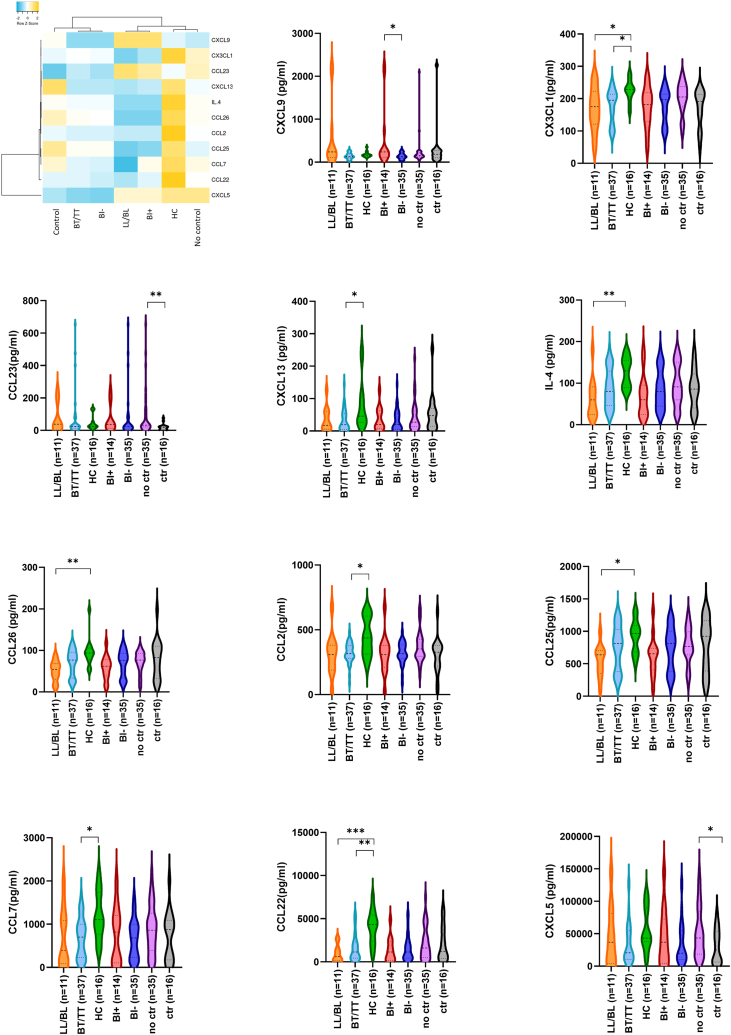
**Cyto-/chemokine levels upon BCG stimulation.** The levels (pg/ml) of 40 cyto-/chemokines upon 96 h stimulation of PBMCs with BCG was determined in lepromatous leprosy patients (LL/BL; n = 12); tuberculoid leprosy patients (BT/TT; n = 37); household contacts of patients with leprosy (HC; n = 16); BI positive patients (BI+; n = 14); BI negative patients (BI−; n = 35); Individuals without (no ctr; n = 35) or with BCG control (ctr; n = 16) *in vitro*. The median cyto-/chemokine level per group of the significant proteins (p < 0.05) was visualised in a clustered heatmap. The heatmap shows clustering based on average linkage performed by heatmapper (Babicki et al., 2016). The z-score indicates the deviation from the average level of the marker across groups, higher Z-scores are indicated in orange and lower Z-scores in blue. Violin plots show the levels (pg/ml; *y*-axis) per group of the significant proteins displayed in the heatmap. Dashed and dotted lines indicate the median and quartiles, respectively. p-values were determined by Mann–Whitney U test (BI+ vs BI− and no control vs control) or Kruskall–Wallis test with Dunn's correction for multiple testing (LL/BL vs HC; BT/TT vs HC and LL/BL vs BT/TT). ∗p < 0.05; ∗∗p < 0.01; ∗∗∗p < 0.001.

The cyto-/chemokine profile of patients and contacts was determined in supernatant after 96 h-stimulation of PBMCs with BCG to assess responsiveness upon mycobacterial stimulation. Most significant differences were observed between patients and contacts (Fig. 6), with higher levels of CCL2, CCL7, CCL22, CCL25, CCL26, CXCL13, CX3CL1, and IL-4 in contacts (n = 12) compared to LL/BL (n = 11) and/or BT/TT (n = 36) patients. In general, a gradient associated with the level of *M. leprae* control can be observed for these proteins, ranging from lowest levels in LL/BL patients, intermediate levels in BT/TT patients, and highest levels in contacts (Fig. 6). Among patients with leprosy, higher CXCL9 levels were observed in patients with high bacillary loads. *In vitro* BCG control was associated with lower levels of CCL23 and CXCL5, showing no overlap with cyto-/chemokines identified for *M. leprae* control. Stimulation with BCG unveiled a strong induction of cyto-/chemokines in contacts compared to patients, indicating the importance of cyto-/chemokine production in response to mycobacterial antigens to combat *M. leprae* infection without developing clinical symptoms.

### Cyto-/chemokine induction in contacts is positively associated with the expression level of CXCR3, CCR4, and HLA-DR

**Fig. 7:**
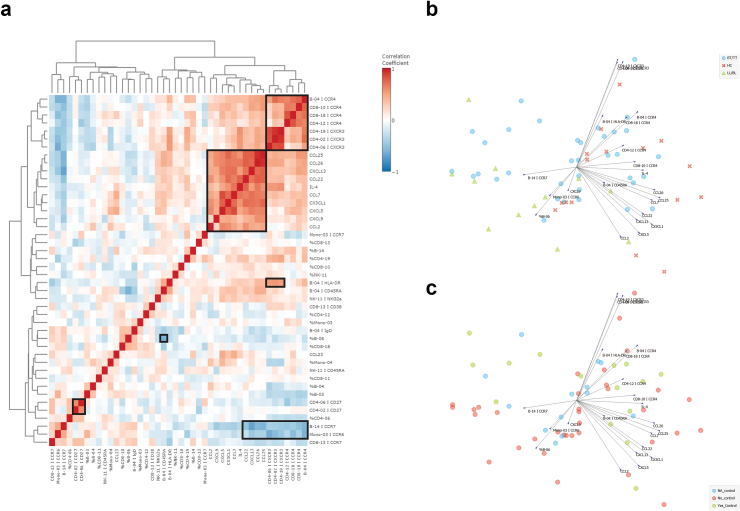
**Integrative analysis of immune cell subsets, marker expression and cyto-/chemokine production.** (a) Correlation heatmap visualising the spearman rank correlation between the significant immune cell subsets (%), MFI and cyto-/chemokine levels (pg/ml). All values were log10 transformed and the spearman rank correlation was determined. Red indicates a positive correlation between two variables and blue a negative correlation. Squares indicate clusters with containing spearman R values >0.5 or <−0.5. (b) Principal component analysis (PCA) biplot of variables indicated in (a). Green triangles: lepromatous leprosy patients (LL/BL; n = 12); blue circles: tuberculoid leprosy patients (BT/TT; n = 37); Red crosses: household contacts of patients with leprosy (HC; n = 16). (c) Principal component analysis (PCA) biplot of variables indicated in (a). Green circles: individuals controlling BCG *in vitro* (n = 16); Red circles: individuals without BCG control *in vitro* (n = 35); *blue circles*: BCG control status was not available for these individuals (n = 13).

To integrate the quantitative and qualitative analysis of immune cell subsets with the induction of cyto-/chemokines upon *in vitro* BCG stimulation, a correlation matrix was computed for all significant results obtained in the analyses above. Except for CCL23, all cyto-/chemokines cluster together showing a significant positive correlation (Fig. 7a). This cyto-/chemokine cluster significantly correlated with CCR4 and CXCR3 expression on CD4+ T, CD8+ T and B cells. The expression of CXCR3 on CD4+ T cells also correlated significantly with HLA-DR expression on subset B-04. Inverse correlation was observed for the CCR4+/CXCR3+ cluster with CCR7 expression on B-14 and CCR6 on mono-03, and for %B-06 (IgD+) with CD45RA expression on B-04 (CCR4+/IgD+). The inverse pattern of positive CXCR3/CCR4/HLA-DR and negative CCR6/CCR7 correlation with the cyto-/chemokine cluster was also observed in the PCA biplot (Fig. 7b). Contacts of patients with leprosy are mainly associated with CXCR3/CCR4/HLA-DR and LL/BL patients with %B-06 (IgD+) and CCR6/CCR7. BT/TT patients are scattered over the PCA biplot, confirming their intermediate phenotype between contacts and LL/BL patients. No clear association between *in vitro* BCG control and this inverse pattern was observed (Fig. 7c).

The here described integrative analysis indicates a significant link between the strong induction of cyto-/chemokines in contacts of patients with leprosy who are able to control *M. leprae* growth *in vivo*, and the expression of HLA-DR, CXCR3, and CCR4 on adaptive immune cells, including B cells, thereby identifying immune cell subsets in leprosy beyond the Th1/Th2 paradigm.

## Discussion

The hallmark of leprosy is its disease spectrum which reflects the ability of individuals to mount protective immunity against *M. leprae*, leading to eradication of the bacterium from the host's tissues. Despite this decade-long knowledge, immune correlates of protection from disease are largely unknown. This not only hampers development of new diagnostics and treatment, but also to monitor the efficiency of personalised prophylactic treatment such as the WHO-endorsed SDR-PEP.^51^ The inability to culture *M. leprae in vitro* impedes functional studies on host immunity in patients with leprosy. Therefore, to identify host factors related to mycobacterial control in patients with leprosy and their contacts *in vitro*, functional MGIAs were performed with a closely related mycobacterium, BCG. Contrary to the spectral presentation of leprosy, no intrinsic differences in BCG growth control *in vitro* between patients lacking the ability to control *M. leprae in vivo* (LL/BL) and patients controlling *M. leprae* growth (BT/TT) and household contacts was observed. Thus, the *in vitro* capacity to control BCG does not parallel with *in vivo* control of *M. leprae*, as also evidenced by the lack of overlap in immune cell subsets associated with control of both mycobacteria. This result indicates specific unresponsiveness to *M. leprae* in LL/BL patients, based on factors beyond the Th1/Th2 paradigm.^52^ CD14^dim^ monocytes were, however, associated with control of both *M. leprae* and BCG. Since these monocytes have previously been shown to correlate strongly with control of BCG growth *in vitro* in individuals exposed to *M. tuberculosis*,^36^ our data confirm the validity of the MGIA as a tool to identify immune cell subsets associated with mycobacterial control.

In the past, studies on leprosy, particularly concerning the differences between its polar forms that are characterised by immunosuppressive states on one hand and overreactive, autoimmune-like T cell immunity on the other hand, have shown that leprosy is an excellent model disease to unravel host immune responses.^20^ These studies demonstrated Th1/Th2 dualism,^22^ HLA-disease association,^24^^,^^25^ and identified human regulatory T cells.^23^ The here performed extensive immunophenotyping is one of the few studies for leprosy with an integrative analysis of all major immune cell subsets in PBMCs of patients and contacts. Extensive analysis of PBMCs using single cell RNAseq has been described for patients with leprosy, contacts and endemic controls in the Chinese population.^53^^,^^54^ However, since patients are sporadically diagnosed in high-income countries,^55^ the challenges to obtain PBMCs in low resource leprosy endemic areas and subsequent use for cutting edge experiments, limit the number of such leprosy studies performed. Moreover, previous leprosy research studies did not combine immunophenotyping with a functional assay on the same cells to assess the antimycobacterial response.

Immunophenotyping demonstrated a similar composition of (unstimulated) PBMCs in patients with low bacillary loads (BT/TT) and contacts. This is in line with previous results, confirming that even though contacts control *M. leprae* growth *in vivo* and do not present disease symptoms, their immune response to *M. leprae* closely resembles that of BT/TT patients.^30^^,^^33^^,^^34^^,^^56^ However, a strong induction of cyto-/chemokines (i.e., CCL22, CCL25, CCL26) after *in vitro* PBMC stimulation with BCG for four days was observed in contacts, confirming the importance of antigenic stimulation to assess the host response to *M. leprae* infection more sensitively.^30^^,^^57^ Cytokine and chemokine levels induced by BCG stimulation were positively correlated with the expression levels of chemokine receptors CCR4 (B and T cells) and CXCR3 (CD4 T cells). CXCR3 has been previously identified as an important chemokine receptor for mycobacterial control.^36^ This receptor for CXCL9, CXCL10, and CXCL11 was expressed on only few CD4+ T cells in patients with high bacterial loads. The particular absence of CXCR3 on CD4+ T cells in these patients unable to control *M. leprae*, indicates that CXCR3 expression could be required for *in vivo* control of *M. leprae*.

Although the chemokine receptor CCR4 was included to identify Th2 cells,^44^ it is expressed on Th17 cells as well.^58^ Its expression is essential for migration of activated T cells into the skin,^59^ which is a primary site of *M. leprae* infection. CCR4 serves as the receptor for CCL17 and CCL22, the latter displaying the most prominent difference in this study between contacts and patients with leprosy, with unprecedented discriminatory potential for BT/TT patients and contacts.^56^ The CCL22-CCR4 axis plays a role in the control of T cell immunity; CCL22-deficient mice had excessive T cell responses after vaccination and increased susceptibility to inflammatory disease.^60^ Moreover, CCR4 has shown to be required to mount an effective Th1 response to mycobacterial antigens in mice.^61^ The high levels of CCL22 observed in contacts compared to patients suggest better control of T cell immunity against *M. leprae*, avoiding excessive (auto)immunity as is the case in the tuberculoid part (BT/TT) of the leprosy disease spectrum. Effective immunity to *M. leprae* in contacts thus seems to be largely dictated by chemokine receptors CXCR3, CCR4, and their ligands.

CXCR3 expression on CD4 T cells was positively correlated with HLA-DR expression on CCR4+ B cells (B-04). HLA-DR is the most prominently present HLA class II molecule, vital for presentation of *M. leprae* antigens to T cells,^62^ and more frequently present in skin lesions of BT/TT patients compared to LL/BL patients.^63^ For leprosy outcome, several HLA-associations have been identified,^24^^,^^64^^,^^65^ displaying its key role in activating antigen-specific immunity. In this study, patients with high bacillary loads expressed significantly less HLA-DR on CCR4+ B cells (B-04) which may result in less presentation of *M. leprae* antigens by these B cells. These results imply an association between *M. leprae*-specific unresponsiveness to mycobacterial antigens and bacterial load, thereby corroborating various earlier reports on downregulation of antigen presentation by *M. leprae*.^32^^,^^66^^,^^67^

In addition to HLA-DR, CD45RA, a marker for naïve B cells,^68^ was also absent primarily on the CCR4+ B-04 subset in patients with high bacterial loads. Interestingly, the expression of HLA-DR/CD45RA on subset B-04 negatively correlated with the percentage of subset B-06. The frequency of the latter IgD-expressing B cells was highest in patients with high bacillary loads, whereas subsets B-03 (CD45RA+) and B-04 (CCR4+) were more abundant in patients presenting with few bacilli. This result shows that B cells are not only involved in the lepromatous pole of the disease but could contribute to control of *M. leprae in vivo* at the tuberculoid pole. Thus, these results challenge the common dogma that B cells are mostly involved in the pathogenesis of lepromatous leprosy. The contribution of B cells to the control of *M. leprae* growth is supported by previous studies, demonstrating B cells participating in the formation of granulomas in biopsies of tuberculoid leprosy patients,^69^ B cells along with *M. leprae*-specific antibody production in lesions of BT patients,^70^ as well as higher bacillary loads in B cell deficient mice experimentally infected with *M. leprae*.^71^

The dogma regarding the role of B cells in lepromatous patients is strongly related to the presence of *M. leprae*-specific antibodies in these patients. Since antigen-specific B cells could not be determined by this study's experimental set-up, it remains to be elucidated whether the IgD+ subset B-06 associated with high bacterial loads plays a role in the generation of *M. leprae*-specific antibodies. In general, mature B cells often express IgD and IgM with identical antigenic specificity. IgD achieves a balance within B cells by attenuating IgM-mediated anergy while promoting the response to non-self-antigens.^72^ IgM directed against PGL-I, a glycolipid specific to *M. leprae*, is detected in high amounts in LL/BL patients. Anti-PGL-I IgM is therefore a useful host biomarker for diagnosis of particularly LL/BL patients, as well as treatment monitoring of this type of patients.^9^, ^10^, ^11^, ^12^, ^13^, ^14^ Anti-PGL-I IgG is often undetectable, and this study confirmed the predominance of IgM in patients, a finding that has also been demonstrated in armadillos experimentally infected with *M. leprae*.^73^ The mechanism of this predominant presence of IgM, as opposed to what is described in textbooks for other infections, is unknown.

A major strength of this study was the performance of extensive immunophenotyping at consecutive levels on PBMC samples processed in low-resource settings, proving feasibility and enabling further advanced studies. For neglected tropical diseases, like leprosy, this is an important step forward, as patient material has to be processed directly in LMICs with limited facilities. A number of limitations can be reported: first, to collect sufficient samples to perform this initial study, it was decided to also include patients with leprosy that had very recently started multidrug therapy. In this dataset, treatment status did not impact the MGIA or immunophenotypic results (data not shown). Secondly, the limited group sizes consisting of patients mainly originating from Bangladesh also affect the statistical power and potential generalisability of the results, requiring replication in different populations. Thirdly, contacts available at the time of sampling were predominantly exposed to BT/TT patients presenting with few bacilli, impeding stratification of contacts exposed to either high or low bacterial loads. Future studies need to address contacts of patients with disseminated disease in more detail, to determine the effect of differential levels of exposure to *M. leprae* on mycobacterial killing and immunophenotypes. Moreover, even though BCG and *M. leprae* are closely related mycobacteria,^43^ they are not antigenically identical and the functional MGIA may not fully reflect the response to *M. leprae*-specific antigens, such as PGL-I. Of note, BCG vaccination provided to neonates in Bangladesh implies that most participants in the study will have some pre-existing immunity to BCG, potentially impacting the control of BCG in the MGIA. However, previous studies have shown that half of those recently vaccinated do not control BCG in the MGIA^41^ and the effect of BCG vaccination on growth control is lost 1-year post-vaccination,^36^ limiting the effect of childhood vaccination on the MGIA results.

To conclude, the functional MGIA applied in the context of leprosy demonstrated a lack of intrinsic difference in *in vitro* mycobacterial control between systemic PBMCs of patients with high and low bacterial loads. Immune cells of LL/BL patients currently perceived as *M. leprae* anergic and immunosuppressive, thus, do not have an intrinsically different killing capacity for BCG compared to BT/TT patients. *In vivo*, however, the integrative analysis demonstrated the unique relation between host immunity and bacillary load, allowing observations beyond the well-defined Th1/Th2 dichotomy between BT/TT and LL/BL patients^8^; B cells associated with either the lepromatous or tuberculoid pole of the spectrum were identified, and CCR4 expression, a classical marker for Th2 cells, was associated with host control of *M. leprae*. Finally, positive correlation of CXCR3/CCR4 levels on adaptive immune cells with a strong induction of cyto-/chemokines in response to mycobacterial antigens in contacts was demonstrated, providing leads to identify immune correlates of protection for leprosy disease.

## Contributors

A.vH., A.G., and K.E.vM. designed the study. A.vH., A.G., and K.E.vM. accessed and verified the underlying data and performed the data analysis. A.vH. and A.G. wrote the manuscript. A.vH. and K.E.vM. performed MGIA and spectral flow experiments. K.W. was responsible for the bacterial cultures. C.L.M.vH., K.A., M.K., S.S., and A.S.C. were responsible for recruitment and sample collection. Samples were processed by A.vH., A.G., M.K., and S.S. A.G. and J.H.R. acquired funding. All authors read and approved the final version of the manuscript.

## Data sharing statement

All data generated or analysed during this study are included in this published article. Any additional information required to reanalyse the data reported in this paper is available from the lead contact upon request.

## Declaration of interests

The authors declare no conflict of interest.
