# Supplementary material for: Integrative immune analysis in patients with leprosy reveals host factors associated with mycobacterial control
Source: eBioMedicine. 2025 Jul 18;118:105855. doi: 10.1016/j.ebiom.2025.105855 (PMC12296519; doi:10.1016/j.ebiom.2025.105855)
Supplement: Supplementary Materials [file mmc2.docx]

**Supplemental material**

**Integrative immune analysis in leprosy patients reveals host factors associated with mycobacterial control**

Anouk van Hooij^1^, Krista E. van Meijgaarden^1^, Marufa Khatun^2^, Santosh Soren^2^, Kimberley Walburg^1^, Khorshed Alam^2^, Abu Sufian Chowdhury^2^, Colette L.M. van Hees*^3^*, Jan Hendrik Richardus*^4^*, and Annemieke Geluk^1^

*^1^ Dept. Infectious Diseases, LUCID, Leiden University Medical Center, The Netherlands*

*^2^ Research Program, The Leprosy Mission International Bangladesh, Nilphamari, Bangladesh*

*^3^ Department of Dermatology, Erasmus Medical Center, Rotterdam, The Netherlands.*

*^4^ Department of Public Health, Erasmus MC, University Medical Center Rotterdam, Rotterdam, The Netherlands*


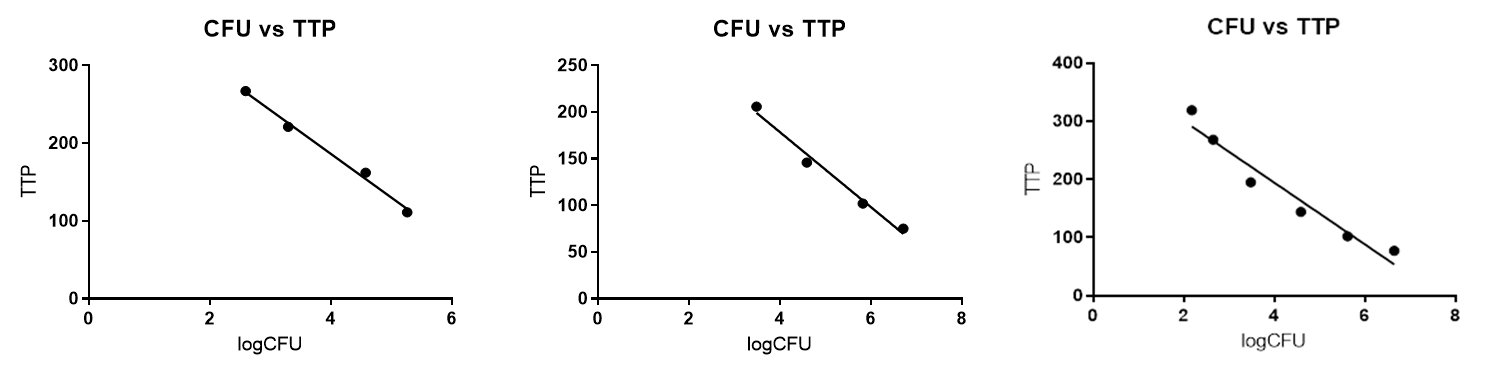


**Supplementary Figure S1: Serial dilution of the BCG stock as a control for the BCG inoculum.** All experiments included a serial dilution (10^7^–10^2^) of the BCG stock for time to positivity in PANTA/Enrichment supplemented MGIT tubes and plating on Middlebrook 7H10 agar plates, supplemented with 10% OADC (BD) for colony forming units (CFU) determination. Plates were scanned on a Canon Scanner 9000 F and colonies were counted using ImageJ software. CFUs were converted to logCFU and plotted against time to positivity (TTP; *y*-axis). Each graph represents the serial dilution for a single experiment.


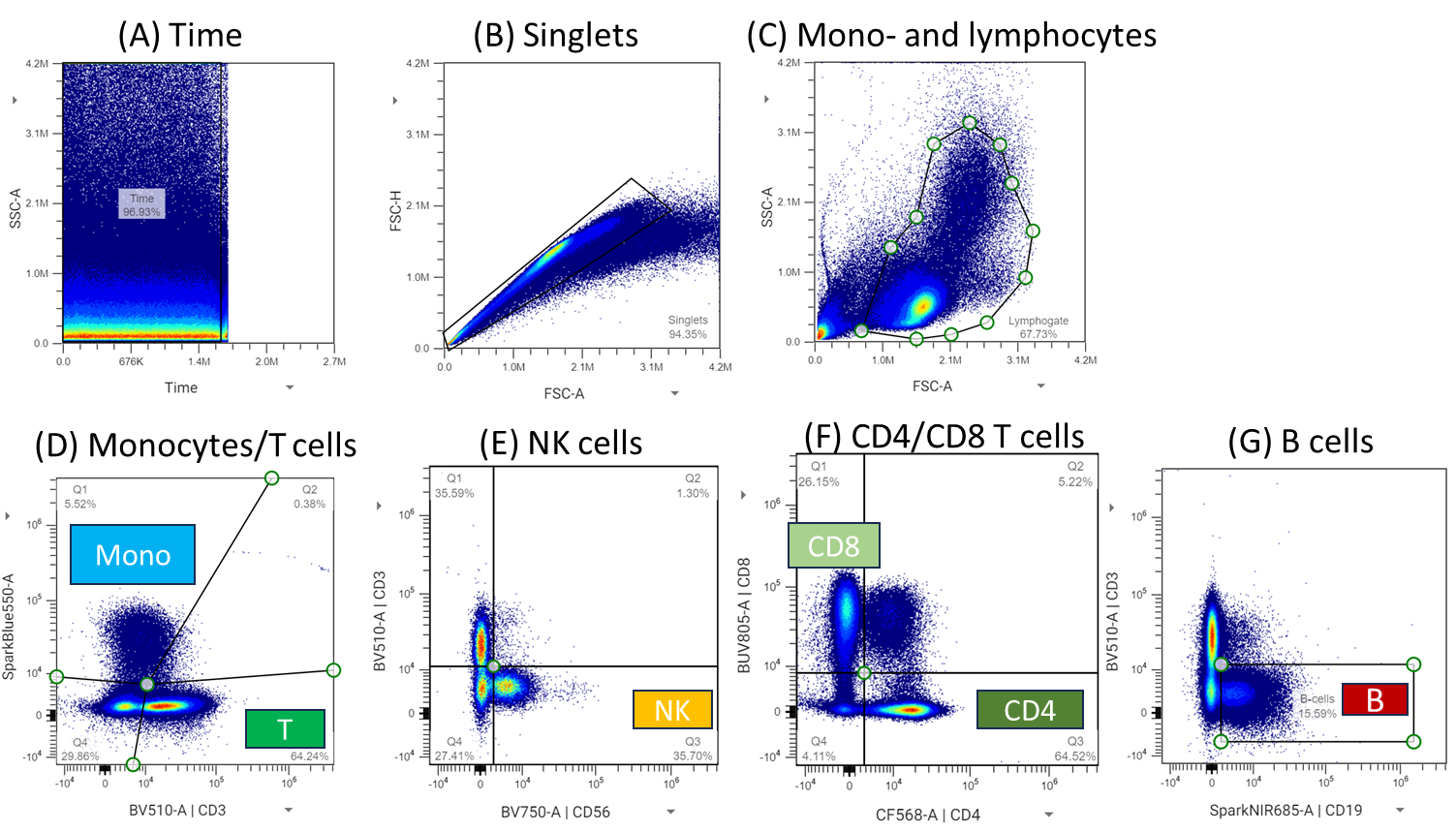


**Supplementary Figure S2: Gating strategy to determine major lineage immune cells.** Datafiles were cleaned by setting a time gate (A) and removal of doublets (B). The monocyte and lymphocyte populations were selected using forward versus side scatter gating (C). Within this gate, monocyte, NK cell, T cell and B cell populations were identified by manual gating. (D) The CD3 -/ CD14 + gate was used to determine the monocyte population and CD3+/CD14- cells as T cells. (E) NK cells were determined as CD3-/CD56+. (F) CD3+ cells were selected and CD4+ and CD8+ T cells were determined within this gate (CD4+/CD8- and CD4-/CD8+ respectively). (G) CD3 -/ CD19+ cells were characterized as B cells.


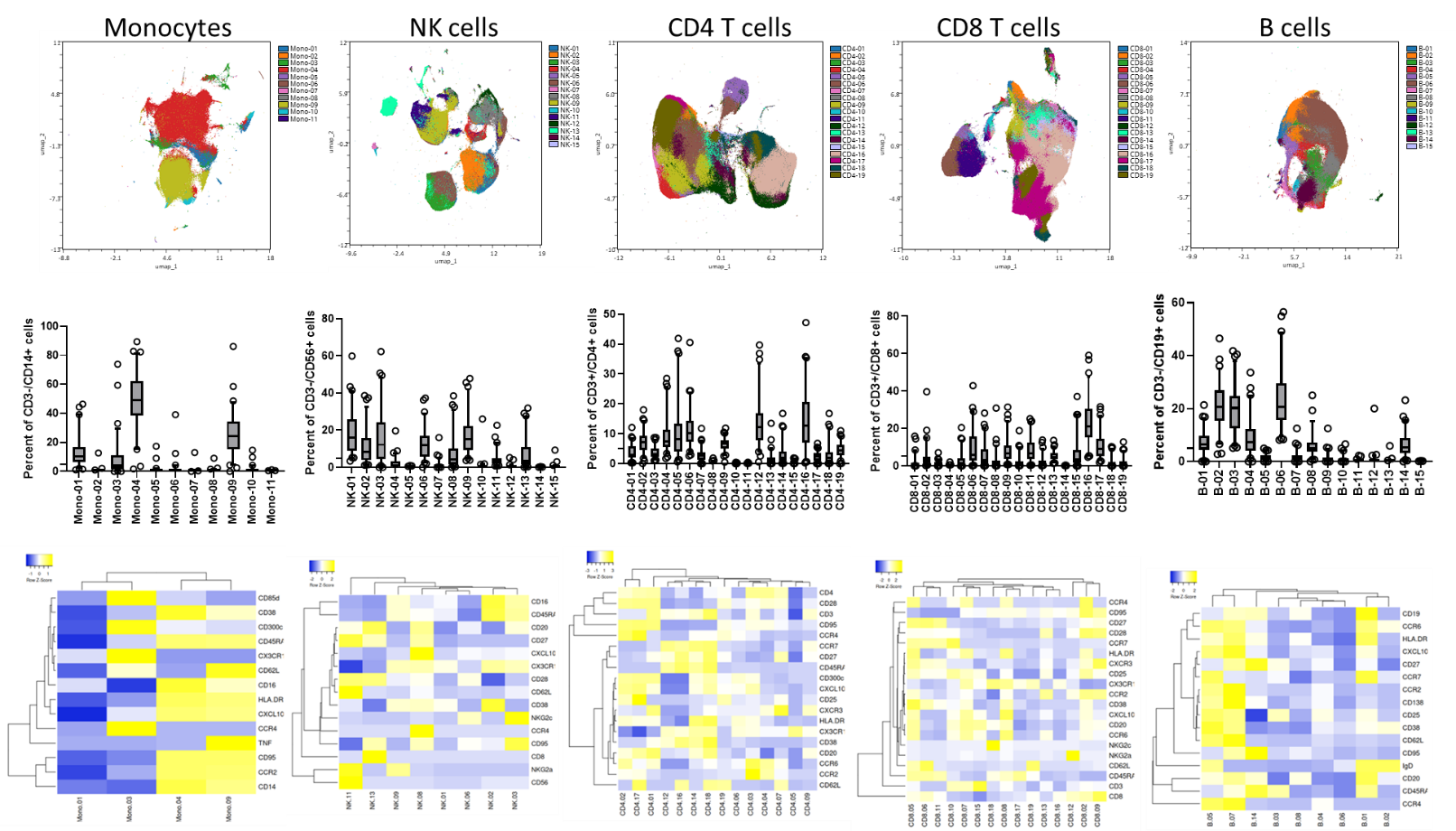


**Supplementary Figure S3:** Unbiased identification of monocyte, NK cell, CD4+ T cell, CD8+ T cell and B cell subsets by UMAP dimension reduction. FlowSOM was applied to identify metaclusters of cells based on the marker expression pattern of the 32-color panel. Top row shows the UMAP plots and identified clusters by elbow metaclustering per major lineage immune cells. This analysis identified 11 monocyte subsets, 15 NK cell subsets, 19 CD4+ T cell subsets, 19 CD8+ cell subsets and 15 B cell subsets. Box plots (middle row; whiskers 5-95 percentile) indicate the percentages per subset for all individuals. Populations >100 cells were included in the analysis and marker expression of these subsets was visualized by clustered heatmaps. Relevant markers for each of the five major lineage immune cell subsets were visualized in the heatmaps: Yellow indicates high expression compared to other subsets; blue corresponds to low expression compared to other subsets (bottom row).


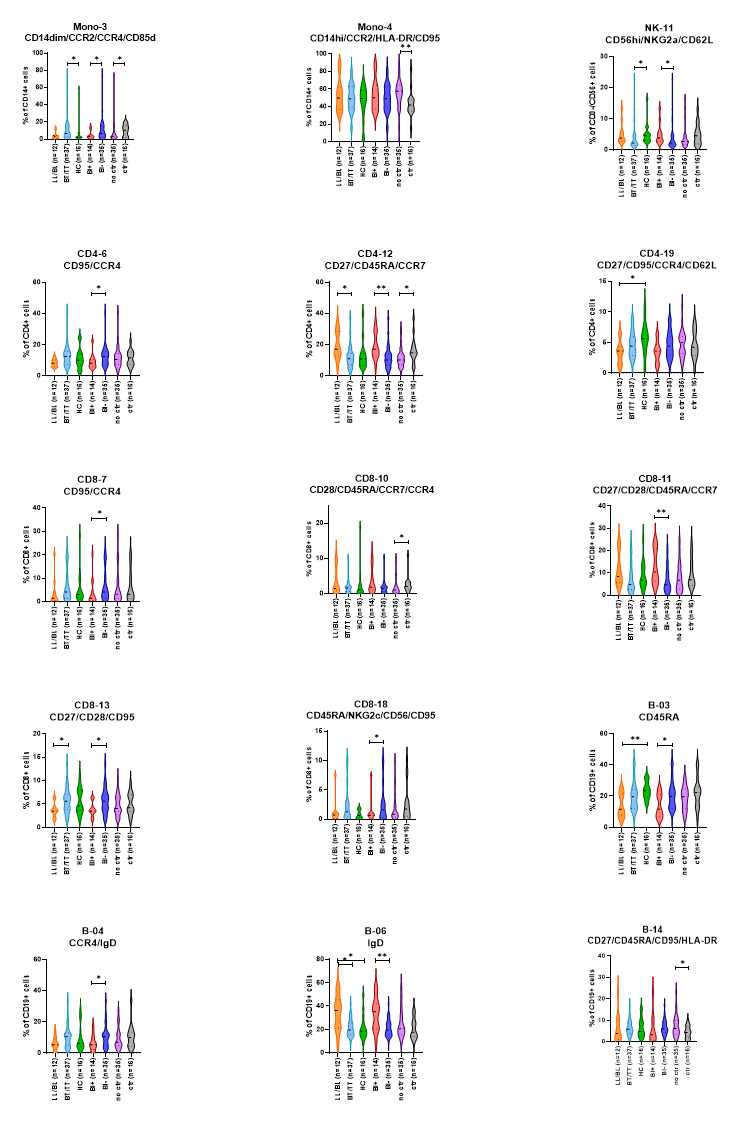


**Supplementary Figure S4:** Violin plots indicating the median (dashed line) and quartiles (dotted line) of percentages of 15 meta-clusters with significant differences between individuals controlling *M. leprae* *in vivo* and/or BCG *in vitro.* lepromatous leprosy patients (LL/BL; n=12) ; tuberculoid leprosy patients (BT/TT; n=37) ; household contacts of leprosy patients (HC; n=16) ; BI positive leprosy patients (BI + ; n=14); BI negative leprosy patients (BI- ; n=35) ; Individuals without (no ctr; n=35) or with BCG control (ctr; n=16) *in vitro*. P-values were determined by Mann-Whitney U test (2 groups) or Kruskall-Wallis test with Dunn’s correction for multiple testing (3 groups). p-values: < 0,05* ; <0,001**


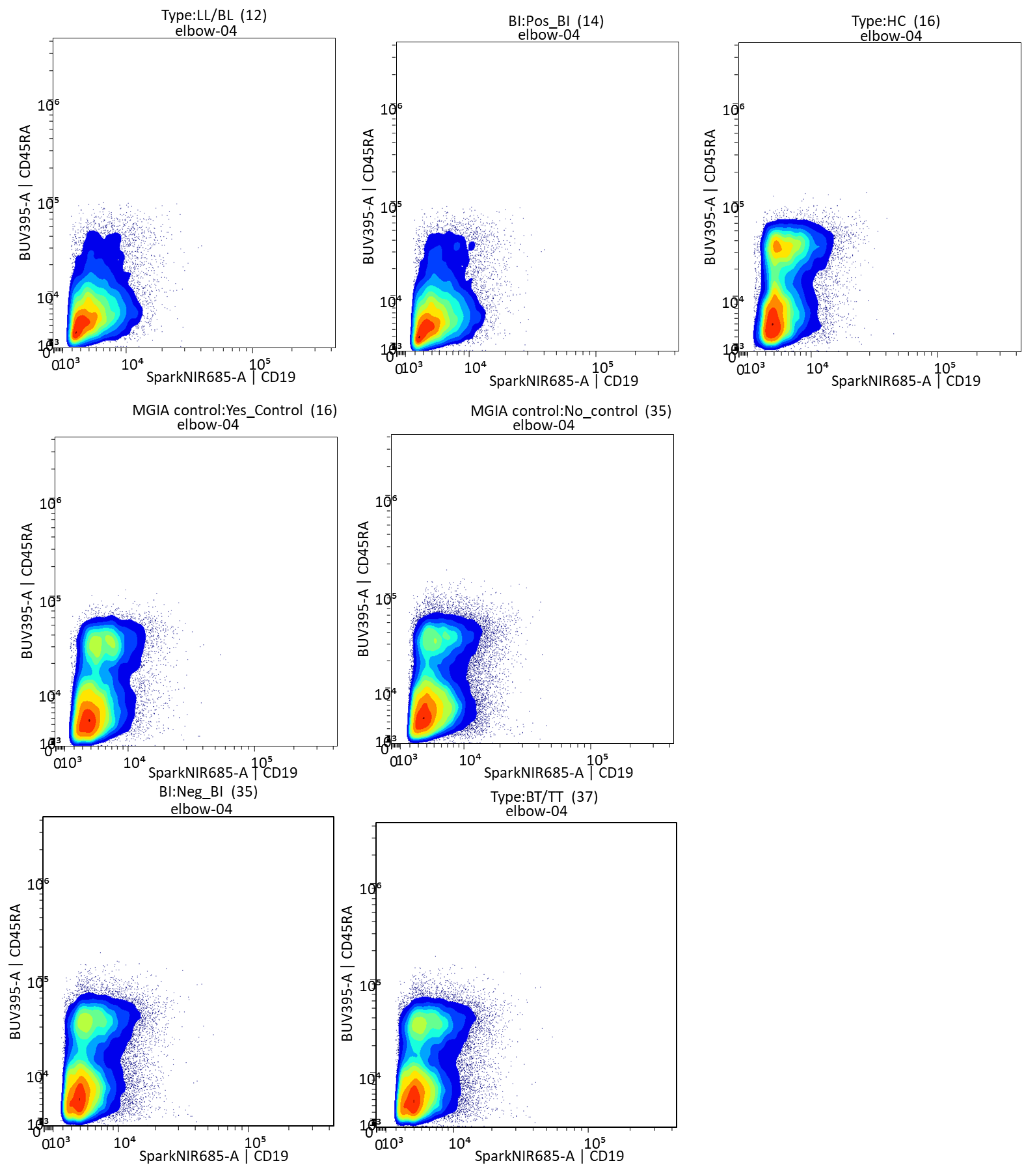


**Supplementary Figure S5:** Concatenated dot plots depicted in figure 2 panel 3 with higher resolution. Concatenated dot plots show the CD45RA expression level (*y*-axis) on subset B-04 (CD19 expression on *x*-axis) stratified per group.

**Table legends**

**Supplementary Table S1:** Overview of leprosy patients (n=46) and household contacts (n=17) recruited for the study. Gender: M = male ; F = female. BI: bacterial index, for contacts the bacterial index of the index case is indicated (italic). Type: classification of patients according to Ridley-Jopling. BT= borderline tuberculoid ; BL =borderline lepromatous ; BB = midborderline. For contacts, classification of the index case is indicated (italic). Treatment: treatment status of patients at moment of sampling. Yes = received multidrug therapy ; No = no treatment received. Duration of treatment: NA = not applicable. Country of sampling: country of sample collection and processing. Relation to index case: relationship of the respective contact with the index case. Anti-PGL-I IgM & IgG: anti-PGL-I IgM and IgG levels determined by ELISA (OD_450_-background). OD_450_-background > 0.200 is considered as a cut-off for positivity (indicated in green). MGIA: was mycobacterial growth inhibition assay (MGIA) performed for this individual (yes/no). MGIA_control: individual was classified based on the control of BCG *in vitro* (MGIA result; logCFU < 2.59 = control). logCFU_MGIA: log colony forming units as determined by MGIA. Spectral flow: was spectral flow cytometry performed to determine the immunophenotype of this individual (yes/no). MBA: Was multiplex bead assay performed on supernatant stimulated for 96 hours with BCG for this individual (yes/no)

**Supplementary Table S2:** Overview of fluorochromes used to stain the markers indicated in the table. Clones of the antibodies used, company catalogue number and RRID are described. The dilution of the antibody and the temperature used to stain the cells is indicated in the final two columns (RT = room temperature).

**Supplementary Table S3:** Mean fluorescence intensity within immune cell subsets compared between groups. The MFI of all markers expressed within subsets was compared in lepromatous leprosy patients (LL/BL; n=12) ; tuberculoid leprosy patients (BT/TT; n=37) ; household contacts of leprosy patients (HC; n=16) ; BI positive leprosy patients (BI + ; n=14); BI negative leprosy patients (BI- ; n=35) ; Individuals without (no control; n=35) or with BCG control (n=16) *in vitro*. P-value was determined by Mann-Whitney U test.
